# Topical antimicrobial treatment of mesh for the reduction of surgical site infections after hernia repair: a systematic review and meta-analysis

**DOI:** 10.1007/s10029-024-02987-0

**Published:** 2024-05-09

**Authors:** Nathan Bontekoning, Nathalie J. Huizing, Allard S. Timmer, Hannah Groenen, Stijn W. de Jonge, Marja A. Boermeester

**Affiliations:** 1 Department of Surgery, Amsterdam UMC Location University of Amsterdam, Meibergdreef 9, Amsterdam, The Netherlands; 2 Amsterdam Gastroenterology Endocrinology & Metabolism, Amsterdam, The Netherlands

**Keywords:** Mesh, Topical, Pretreatment, Antimicriobials, Antibiotics, Surgical site infection

## Abstract

**Purpose:**

Use of mesh is essential in hernia repair. A common complication after hernia repair is surgical site infection (SSI), which poses a risk in spreading to the mesh, possibly causing mesh infection. Topical antimicrobial pretreatment of mesh may potentially reduce SSI risk in hernia repair and has shown promising results in in vitro and in vivo studies. Clinical evidence, however, is more important. This systematic review aims to provide an overview of available clinical evidence for antimicrobial pretreated mesh in hernia repair surgery to reduce SSI.

**Methods:**

We report in accordance with PRISMA guidelines. CENTRAL, EMBASE, CINAHL and PubMed were searched up to October 2023 for studies that investigated the use of antimicrobial pretreated mesh on SSI incidence in adults undergoing hernia repair. The primary outcome was SSI incidence. We also collected data on pathogen involvement, hernia recurrence, and mesh infection. A meta-analysis on SSI risk and GRADE-assessment was performed of eligible studies.

**Results:**

We identified 11 eligible studies (*n* = 2660 patients); 5 randomized trials and 6 cohort studies. Investigated interventions included pre-coated mesh, antibiotic carriers, mesh soaked or irrigated with antibiotic or antiseptic solution. Meta-analysis showed no significant reduction in SSI for antibiotic pretreated polypropylene mesh (RR 0.76 [95% CI 0.27; 2.09]; *I*^2^ 50%).

**Conclusion:**

Data on topical mesh pretreatment to reduce SSI risk after hernia repair is limited. Very low certainty evidence from randomized trials in hernia repair surgery shows no significant benefit for antibiotic mesh pretreatment for SSI reduction, but data are imprecise due to optimal information size not being met.

**Supplementary Information:**

The online version contains supplementary material available at 10.1007/s10029-024-02987-0.

## Introduction

Hernia repair surgery impacts over 20 million people worldwide yearly [1]. Surgical site infection (SSI) remains one of the biggest challenges within the hernia repair field, leading to increase in morbidity, mortality and costs [2]. Over the years, different methods and techniques have been developed to improve surgical outcomes and minimize the occurrence of postoperative complications, such as SSI.

The development and use of mesh prosthetics has had great impact on the field of hernia surgery [3]. These mesh, available in a large variety of compositions (i.e., biologic, various synthetic components), can enhance the structural integrity of the hernia repair, providing stability and reinforcement to the abdominal wall. The incorporation of surgical mesh into hernia repair procedures, both in groin and ventral/incisional hernias, has led to reduced hernia recurrence rates [4]. However, concerns regarding the risk of SSI still remain relevant, as the use of prosthetic materials provides an opportunity for bacteria to attach to the surgical mesh and develop biofilms [5]. These infections are often caused by bacteria that are naturally present in the skin flora, including *Staphylococcus aureus, Staphylococcus epidermis, Escherichia coli, and Enterococcus species* [6]*.*

In response to these concerns, treatment with intravenous antibiotic prophylaxis is predominantly used to mitigate infection rates [7]. Currently, there is a substantial body of research dedicated to systemic antibiotic prophylaxis. Its use has become standard of care in open ventral hernia repairs, while the efficacy for groin hernia surgery remains equivocal, raising concerns about its systemic side effects [8]. Recently, there has been a focus shift toward antimicrobial properties of the surgical mesh itself, exploring the incorporation of various antimicrobial compounds, such as antimicrobial metals (e.g., silver, titanium, zinc and gold), antiseptics (e.g., povidone-iodine or chlorhexidine) and antibiotics (e.g., ampicillin, gentamicin, cefazolin, rifampicin, minocycline) [9]. This exploration includes various techniques, such as soaking, irrigation, coating, and impregnation. Various in vivo and in vitro studies show promising results [9].

Despite valuable insights provided by these experimental studies on the potential benefits of antimicrobial mesh treatment, there remains a scarcity of clinical studies that assess the effectiveness of this approach. This review aims to gather the current clinical evidence that is available and evaluate the impact of antimicrobial pretreated mesh on SSI in hernia repair surgery.

## Methods

### Search strategy and study selection

We report according to the Preferred Reporting Items for Systematic Reviews and Meta-Analyses [10]. The study protocol is available on the PROSPERO database (CRD42023471619).

All clinical, published and unpublished studies investigating the effects of antimicrobial-treated mesh on SSI in adult human patients were eligible for inclusion. Only randomized controlled trials (RCTs) were considered for pooling in meta-analysis. Studies before the year 2000 were excluded, because they most likely did not utilize the most recent standards in perioperative clinical care, as described by Mangram et al. [11]. There was no restriction on language.

The Medline (PubMed), EMBASE (Ovid), CINAHL (EBSCO) and Cochrane Central Register of Controlled Trials (CENTRAL) databases were searched up to October 24, 2023. Key search terms included::“Hernia”, “Surgical procedures, Operative”, “Surgical Mesh”, “Anti-Bacterial Agents”, “Antiseptic”, “Infections” and “Surgical wound infection”. Any additional articles were unearthed through cross-referencing. The complete search strategy can be found in Online Resource 1. Full- text review and assessment were carried out when the title and abstract screening indicated the study eligibility.

### Data collection and analysis

Data were extracted according to a pre-defined data abstraction form by two reviewers (NB and NH) independently. Study characteristics that were extracted included: study design, sample size, primary outcome, secondary outcome(s), route and type of agent used on mesh, mesh type, mesh location, type of surgery, wound classification as defined by the Centers for Disease Control and Prevention (CDC) [12]. Outcome data included incidence and definition of SSI (i.e., superficial, deep and organ space), hernia recurrence rate, follow up, reported pathogens and mesh infection. In case of missing data on SSI incidence, the corresponding authors were contacted.

The revised Cochrane risk of bias tool (RoB 2) was used to assess the risk of bias in the RCTs. Observational studies’ quality was judged with the Newcastle–Ottawa quality assessment form. Screening, data extraction, and bias/quality assessment were performed independently by two reviewers (NB and NH). Discrepancies were resolved through discussion.

Study characteristics are presented descriptively. If appropriate, outcome data were summarized in meta-analysis.

Relative risk (RR), corresponding 95% CI and standard errors were calculated for the individual comparative trial arms. Only RCTs with comparable administration of systemic antibiotic prophylaxis in either arm were pooled in the quantitative analysis, due to its strong effect on the primary outcome. Meta-analysis was performed using a random-effects model (Mantel-Haensel). A *p* value < 0.05 was considered statistically significant. Statistical heterogeneity was assessed using the *I*^*2*^.

The Grading of Recommendations, Assessment, Development and Evaluations (GRADE) approach was used for rating certainty of evidence using a minimally contextualized approach on the following domains: risk of bias, inconsistency, indirectness, imprecision, and publication bias [13]. The minimally important difference was defined as 1.15% based on the default for appreciable benefit and harm of 25% and the SSI incidence of 4.6% in data of present meta-analysis of included RCTs for patients without antibiotic mesh treatment [14]. Inconsistency was assessed using *I*^2^ and *τ*^2^ statistics [15]. An *I*^2^ < 25% is considered as low, between 25 and 50% is considered moderate, and > 50% as high. We evaluated imprecision taking the minimally important differences into account. In case of large effects, the optimal information size approach was used by calculating the ratio of the upper to the lower boundary of the confidence interval with a threshold for downgrading of 2.5 [16].

Quantitative analyses were done using R, version 4.2.1 [R Core Team (2016) R: A language and environment for statistical computing; R Foundation for Statistical Computing, Vienna, Austria], using the packages meta, metaphor and tidyverse.

## Results

### Study selection

**Fig. 1:**
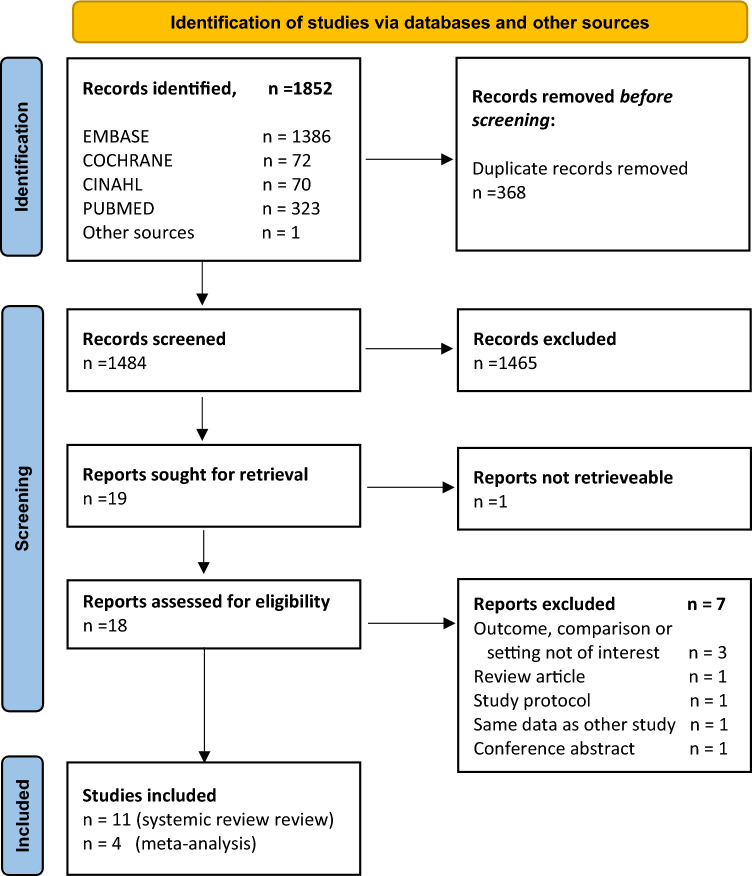
PRISMA systematic review flow diagram

The PRISMA flowchart for study selection is shown in Fig. 1. Our search identified 1852 studies. After full-text screening of 18 articles, 11 were included in the systematic review. Reason for exclusion after full-text screening per article are presented in Online Resource 2.

### Study characteristics

Study characteristics are presented in Table 1 and Online Resource 3. We included five RCTs [17–21], one comparative prospective study [22], one comparative retrospective study [23], three prospective cohort studies [24–26] and one retrospective cohort study [27]. Notably, two studies had three study arms each [18, 22]. Studies were published between 2001 and 2023. Seven studies investigated incisional or ventral hernia repairs and four studies describe patients undergoing inguinal hernia repair. All studies described the use of an antibiotic agent in the pretreatment of the mesh. Only one study by Schneeberger et al. 2020 [26] incorporated an antiseptic (povidone-iodine), in combination with antibiotics, for mesh pretreatment. No studies investigated the use of antimicrobial metals. Contamination levels varied.

**Table 1:** Study characteristics of included studies

Study design	Author, year	Type of mesh treatment	SAP	SSI/n	Type of mesh	Type of surgery	CDC-WC	Follow up	Cochrane risk of bias	Included in meta-analysis
RCT	Musella (2001)	Gentamicin collagen tampon^a^	Yes	1/293 (0.3%)	Polypropylene	Inguinal hernia repair	I	180 days	Some concerns	Yes
		No antimicrobial mesh treatment	Yes	6/284 (2.1%)						
	Praveen (2009)	Soaked in gentamicin	No	8/100 (8%)	Polypropylene	Open inguinal hernia repair	I	30 days	Some concerns	No^b^
		No antimicrobial mesh treatment	Yes	7/102 (6.9%)						
	Şeker (2021)	Topical gentamicin	No	2/87 (2.3%)	Polypropylene	Open inguinal hernia repair	I	30 days	High	Partially^b^, 2 groups
		Topical gentamicin	Yes	0/91 (0%)						
		No antimicrobial mesh treatment	Yes	3/98 (3%)						
	Yabanoğlu (2015)	Soaked in vancomycin	Yes	8/26 (30.7%)	Polypropylene	Open ventral hernia repair	I	4 months	Some concerns	Yes
		No antimicrobial mesh treatment	Yes	4/26 (15.4%)						
	Warren (2023)	Irrigated with gentamicin + clindamycin	Yes	10/110 (9.1%)	Polypropylene	Open ventral hernia repair	I–IV	30 days	Some concerns	Yes
		No antimicrobial mesh treatment	Yes	11/111 (9.9%)						
Observational									NOS	
Comparative	Fatula (2018)	Irrigated with gentamicin	Yes	40/263 (15.2%)	Synthetic and Biologic	Open ventral hernia repair	I–IV	NR	Poor	No
		Irrigated with gentamicin + clindamycin	Yes	16/299 (5.4%)						
		No antimicrobial mesh treatment	Yes	43/260 (16.5%)						
	Kahramanca (2013)	Topical rifampicin	No	6/134 (4.5%)	Polypropylene	Inguinal hernia repair	I	6 months	Poor	No
		No antimicrobial mesh treatment	No	16/144 (11.1%)						
Single arm	Baker (2016)	Coated with rifampicin + minocycline	NR	5/74 (6.8%)	Biologic	Open and laparoscopic abdominal wall hernia repair	I–IV	6 months	Poor	No
	Drohan (2020)	CSAB with vancomycin + gentamicin^a^	Yes	2/11 (18.2%)	Biologic	Incisional ventral hernia repair	II–IV	24 months	Poor	No
	IIahi (2023)	Coated with rifampicin + minocycline	Yes	4/59 (6.8%)	Biologic	Open ventral hernia repair	I–IV	24 months	Poor	No
	Schneeberger (2020)	Soaked in povidone iodine + bacitracin + gentamicin + cefazolin	NR	4/88 (4.5%)	Synthetic	Open ventral hernia repair	I–III	365 days	Poor	No

^a^Placed in front of the mesh

^b^Trial or trial arm did not consistently use systemic antibiotic prophylaxis for all groups, therefore not included in the meta-analysis

*CDC-WC* Centers for Disease Control and Prevention Wound Classification, *CSAB* Calcium Sulfate Antibiotic Beads, *NR* Not Reported, *NOS* Newcastle Ottawa Scale, *RCT* Randomized Controlled Trial, *SAP* Systemic Antibiotic Prophylaxis, *SSI* Surgical Site Infection

Out of the five RCTs included in this review [17–21], four RCTs [17, 18, 20, 21] were pooled in meta-analysis on the efficacy of antibiotic mesh pretreatment on SSI reduction. Data were not included from a trial when systemic antibiotic prophylaxis was not used as a standard: one RCT administered systemic antibiotic prophylaxis exclusively in one group and only topical gentamicin on the mesh in the other arm [19]; similarly, one of the groups in the study by Seker et al. [18] received only topical gentamicin but no administration of systemic antibiotic prophylaxis.

### Mesh treatment characteristics

A global overview of mesh type and treatment are presented in Table 1. A comprehensive description of the antimicrobial mesh treatment (i.e., concentrations of antimicrobials and specifics on topical application), location of mesh placement and systemic antibiotic prophylaxis are listed in Online Resource 4. Seven studies [17–21, 23, 26] used synthetic mesh, with six using polypropylene mesh and one only describing ‘a synthetic mesh’. Three studies [24, 25, 27] exclusively used biologic mesh. Fatula et al. [22] included patients treated with either biologic or synthetic mesh, wherein 3.8% of the patients were treated with a biologic mesh. Overall, 93.4% (2485/2660) of the total study population was treated with a polypropylene/synthetic mesh.

All of the meshes in intervention groups were treated with antibiotics; only one study combined antibiotics and an antiseptic [26]. Six studies [17–20, 22, 23] had an intervention arm wherein the mesh was treated with a singular antibiotic agent. Gentamicin was used in three RCTs and one observational study [17–19, 22]; the other two studies used vancomycin (RCT) [20] and rifampicin (observational) [23]. Mesh were treated with a combination of two antibiotics in one of the arms of five studies [21, 22, 24, 25, 27]; the combinations that were used comprised of gentamicin + clindamycin in one RCT and one observational study [21, 22], and rifampicin + minocycline [25, 27] or gentamicin + vancomycin [24] in observational studies. As mentioned before, the observational trial by Schneeberger et al. 2020 [26] not only treated the mesh with antibiotics but also used an antiseptic; in total, four antimicrobial agents were used for mesh treatment in that specific study (povidone-iodine, bacitracin, gentamicin, cefazolin).

In addition to the diversity of antimicrobials employed, topical mesh treatment techniques varied. The most common method was soaking of the mesh in antimicrobial solution before implantation (two RCTs, one observational study) [19, 20, 26]. Only the RCTs by Yabanoğlu [20] and Praveen [19] specified the time (15 min and 5 min, respectively) of soaking. One RCT and one observational study [18, 23] reported topical application of antibiotics on the mesh without further elaboration. Another RCT and observational study [21, 22] irrigated the mesh with antibiotic solution in the surgical field, maintaining a dwell time of 3 min maximum. Two observational studies [25, 27] used an antibiotic pre-coated surgical polypropylene mesh. In addition, two studies [17, 24] used an antibiotic carrier, which was implanted on top of the mesh before closing; calcium sulfate antibiotic beads with vancomycin + gentamicin (observational) [24] and an absorbable collagen tampon with gentamicin (RCT) [17].

### Quality assessment

The assessment for risk of bias in the RCTs showed ‘some concerns’ for four studies [17, 19–21]. One study [18] was judged as having a high risk of bias. The observational studies were all rated as ‘poor’ with scores ranging from five to seven stars. The full Cochrane Risk of Bias assessment and Newcastle-Ottowa ranking are shown in Online Resource 5.

### Data analysis

Across 11 studies involving 2660 patients, 196 SSI were reported leading to an overall incidence of 7.4%. Incidence ranged from 1.2% to 23.1% among studies. The meta-analysis of 1039 patients with 43 SSIs (4.1%) in the four RCTs comparing topical antibiotic mesh pretreatment with no antibiotic mesh pretreatment showed no significant reduction in SSI (RR 0.76 [95% CI 0.27; 2.09]). Only polypropylene meshes were included in the meta-analysis. With an *I*^*2*^ of 50% statistical heterogeneity was moderate. From all comparative (randomized or observational) studies, only the group from the study by Fatula et al. [22] using mesh pretreatment with both gentamicin and clindamycin solution showed a significant SSI reduction.

**Fig. 2:**
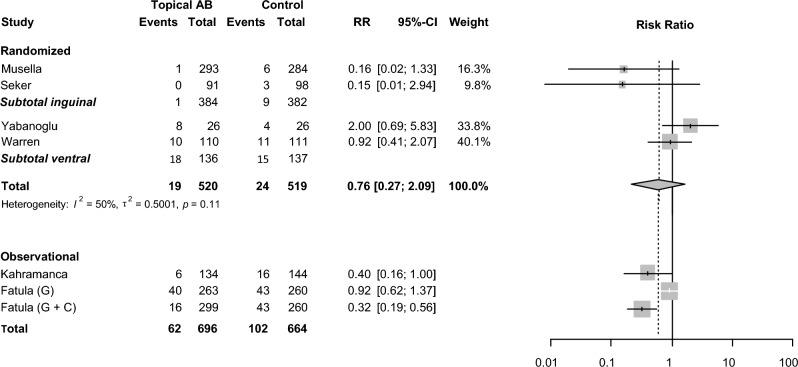
Forest plot of SSI rate in RCTs in which antibiotic mesh treatment is compared with no antibiotic mesh treatment. RCTs with systemic antibiotic prophylaxis administered in both arms are pooled in meta-analysis. Observational studies are not pooled

Data of the comparative observational studies were not pooled because of high heterogeneity. The forest plot for the meta-analysis of RCTs and non-pooled data of comparative observational studies is shown in Fig. 2.

### Certainty of evidence

GRADE assessment, using a minimally contextualized approach, resulted in a very low certainty of evidence, as shown in Table 2. Since all included studies are RCTs, the starting certainty of evidence was high. There were no limitations regarding risk of bias since the result of the sensitivity analysis excluding high risk of bias studies was comparable to the main analysis. For inconsistency, we downgraded one level since heterogeneity was moderate (*I*^2^ = 50%). There was no indirectness [28]. We downgraded two levels for imprecision because the confidence interval overlapped thresholds of interest. For publication bias, rating down one level was necessary because the evidence consists of a number of small studies [29]. In total, we downgraded four levels resulting in a very low certainty of evidence. The full evaluation of certainty of evidence and considerations for grading is shown in Online Resource 6.

**Table 2:** GRADE assessment

	Certainty assessment							No of patients		Effect		Certainty
	No of studies	Study design	Risk of bias	Inconsistency	Indirectness	Imprecision	Publication bias	Topical AB	Control	Relative (95% CI)	Absolute (95% CI)	
SSI	4	RCT	Not serious	Serious (−1 downgrade)	Not serious	Serious (−2 downgrade)	Serious (−1 downgrade)	19/520 (3.7%)	24/519 (4.6%)	0.76 (0.27–2.09)	10 fewer per 1000 (from 15 fewer to 34 more)	⨁◯◯◯ very low

*AB* antibiotics; *CI* confidence interval, *GRADE* Grading of Recommendations, Assessment, Development and Evaluation, *PICO* population, intervention, comparison and outcomes, *RCT* randomized controlled trial, *RR* relative risk, *SSI* surgical site infection

### Secondary outcomes

All secondary outcomes are listed in Online Resource 3. Only five studies [19, 23–25, 27] reported hernia recurrence. Due to the lack of a control group in most of these studies, no quantitative analyses were performed. The same is true for mesh infection, which was only reported by two studies [25, 26] with three cases in total.

Three studies [19, 20, 23] reported on pathogens cultured from wounds. The most found bacterium was *Staphylococcus aureus.* The other bacteria identified in these studies were *Enterobacter, Pseudomonas aeruginosa, Enterococcus faecalis, Proteus mirabilis, Escherichia coli* and* Staphylococcus epidermis.*

## Discussion

This systematic review and meta-analysis aimed to evaluate the effect of antimicrobial-treated mesh on SSI following hernia repair, offering new summary data on this topic. Analysis of 1039 patients with 43 SSIs from 4 randomized trials shows no significant benefit in SSI reduction for antibiotic mesh pretreatment when compared to no antibiotic mesh pretreatment. However, one observational study indicates a benefit of topical antibiotic mesh pretreatment on the risk of SSI.

SSI has the potential to develop into a mesh infection, one of the most detrimental complications of hernia repair [6]. Biofilm formation emerges as a key contributor to SSI and subsequent mesh infections [30], impairing host immune cells and impeding their ability to effectively combat and eliminate bacteria [31, 32]. In general, microorganisms exhibit a tendency to attach to surgical meshes, favoring rough, hydrophobic, and nutritional surfaces, such as polypropylene [31]. Nonetheless, in vitro studies have shown that biologic mesh might be more prone to bacterial adhesion than its synthetic counterpart [29, 31]. Considering the diverse array of available meshes, each differing in structure, composition, weight, porosity, absorbability, and other characteristics, it becomes evident that these variations significantly influence their susceptibility to infection. Therefore, exploring the influence of antimicrobial mesh pretreatment should be coupled with an understanding of the specific mesh type employed. This consideration is crucial when delving further into investigations regarding their collective impact on the risk of SSI and mesh infection.

Our review aimed to explore the clinical evidence regarding all types of antimicrobial mesh pretreatments for reducing SSI. Remarkably, all the studies we examined focused on the use of antibiotics as the primary antimicrobial agent. The sole exception was a study that investigated the topical application of povidone-iodine on mesh; however, even in this case, the antiseptic was combined with three types of antibiotics. Our search yielded no additional literature on the clinical application of antiseptic agents for this purpose.

Given the ongoing antibiotic resistance crisis [33], it is imperative to initiate clinical trials that investigate the potential efficacy of topical antiseptics and metals in the realm of mesh applications. Notably gentamicin, which is extensively used for mesh pretreatment in the included studies in this review, has been implicated in contributing to the escalating resistance observed among Staphylococcus species [34]. In contrast, both povidone-iodine and chlorhexidine as alternative agents considered for mesh pretreatment have not demonstrated a decline in bacterial sensitivity [35, 36].

Interestingly, the RCTs by Praveen [19] and Seker [18] found that topical antibiotic mesh pretreatment, in the absence of systemic prophylaxis, resulted in marginally superior outcomes for SSI compared to systemic antibiotic prophylaxis only. This observation raises considerations for inguinal hernia repair in clean settings, suggesting that the potential benefits of topical therapy may outweigh those of systemic approaches [37].

This review is limited by the lack of high-quality studies and significant clinical heterogeneity of available data. While all included studies incorporated antibiotic mesh pretreatment, substantial variations were observed in the types of antibiotics used, their modes of application, and exposure time. The type of mesh used differed among studies, for example synthetic or biologic mesh. Moreover, mesh was placed in various locations/layers of the abdomen. As mentioned, mesh type has its mesh-specific risk of (mesh) infection. The location for mesh placement is known to be associated with a location-specific risk of SSI and, for example, retro-muscular meshes have better mesh ingrowth [38]. These variables affect the risk of development of mesh infection and thereby the relative effect of mesh pretreatment. Some studies did not report a definition for SSI or worked with other definitions than those outlined in the CDC criteria [12]. In addition, the inclusion of all types of hernia surgery (both inguinal and ventral) introduces the limitation of data scattered across specific populations with varying SSI risk. However, we deem pooling SSI data from these repairs justified, since there is no plausible biological reasoning for effect modification between types of hernia surgery.

In light of the control group’s incidence in our meta-analysis (4.6%), a sample exceeding 10,000 patients would be necessary to demonstrate a clinically relevant 25% reduction in SSI. However, the RCTs included in our analysis, combining for only 1039 patients total, did not (adequately) describe their sample size calculation and are underpowered.

## Conclusions

Data on topical mesh pretreatment to reduce SSI risk after hernia repair is limited. Very low certainty evidence from randomized trials in hernia surgery shows no significant benefit for antibiotic mesh pretreatment for SSI reduction, but data are imprecise due to optimal information size not being met. The diversity in mesh types, modes of antimicrobial agent delivery, and variations in reporting standards have contributed to a challenging landscape for drawing comprehensive conclusions.

### Supplementary Information

Below is the link to the electronic supplementary material.

Supplementary file1 (DOCX 14 KB)

Supplementary file2 (DOCX 15 KB)

Supplementary file3 (DOCX 18 KB)

Supplementary file4 (DOCX 19 KB)

Supplementary file5 (DOCX 87 KB)

Supplementary file6 (DOCX 25 KB)

## Data Availability

All data generated or analyzed during this study are included in this published article.

## Declarations

### Data transparency

NB and NJH had full access to all the data in the study and take responsibility for the integrity of the data and the accuracy of the data analysis.

### Other disclaimers

The information reported in the manuscript has not been presented previously.
