# Supplementary material for: Topical antimicrobial treatment of mesh for the reduction of surgical site infections after hernia repair: a systematic review and meta-analysis
Source: Hernia. 2024 May 9;28(3):691–700. doi: 10.1007/s10029-024-02987-0 (PMC11249405; doi:10.1007/s10029-024-02987-0)
Supplement: Supplementary file 1 — Supplementary file1 (DOCX 14 KB) [file 10029_2024_2987_MOESM1_ESM.docx]

**Topical Antimicrobial Treatment of Mesh for the Reduction of Surgical Site Infections after Hernia Repair**

A Systematic Review and Meta-Analysis

**Hernia**

# **Online Resource 1. Search strategy**

**Medline (via PubMed)**1. "Hernia"[Mesh] OR hernia*[tiab]
2. "Surgical Procedures, Operative"[Mesh] OR "surgery" [Subheading] OR repair*[tiab] OR surg*[tiab] OR surgical*[tiab]
3. "Surgical Mesh"[Mesh] OR mesh*[tiab]
4. "Anti-Infective Agents"[Mesh] OR "Anti-Infective Agents" [Pharmacological Action] OR "Anti-Bacterial Agents" [Pharmacological Action] OR antibiotic*[tiab] OR antibacterial*[tiab] OR anti-bacterial*[tiab] OR anti-infective*[tiab] OR antiinfective*[tiab] OR antiseptic*[tiab]
5. "Infections"[Mesh] OR "Surgical Wound Infection"[Mesh] OR infection*[tiab] OR SSI[tiab] OR SSIs[tiab]

**EMBASE**

1. exp hernia/

2. hernia*.ti,ab,kf.

3. 1 or 2

4. exp surgery/

5. surgery.fs.

6. (repair* or surg* or surgical*).ti,ab,kf.

7. 4 or 5 or 6

8. exp surgical mesh/

9. mesh*.ti,ab,kf.

10. 8 or 9

11. exp antiinfective agent/

12. (antibiotic* or antibacterial* or anti-bacterial* or antiinfective* or anti-infective* or antiseptic*).ti,ab,kf.

13. 11 or 12

14. exp infection/ or surgical infection/

15. (infection* or SSI or SSIs).ti,ab,kf.

16. 13 or 14

17. 3 and 7 and 10 and 13 and 16

18. limit 17 to conference abstract status

19. 17 not 18

**Cochrane CENTRAL**

1. (hernia*):ti,ab,kw

2. (repair* or surg* or surgical*):ti,ab,kw

3. (surgical mesh or mesh*):ti,ab,kw

4. (antibiotic* or anti-bacterial* or antibacterial* or anti-infective* or antiinfective* or antiseptic*):ti,ab,kw

5. (infection* or SSI or SSIs):ti,ab,kw

6. #1 and #2 and #3 and #4 and #5 in Cochrane Reviews, Trials

**CINAHL**

1. (MH "Hernia+") OR TI hernia* OR AB hernia*

2. (MH "Surgery, Operative+") OR TI ( repair* or surg* or surgical* ) OR AB ( repair* or surg* or surgical* )

3. (MH "Surgical Mesh") OR TI mesh* OR AB mesh*

4. (MH "Antiinfective Agents+") OR TI ( antibiotic* or anti-bacterial* or anti-infective* or antibacterial* or antiinfective* or antiseptic*) OR AB ( antibiotic* or anti-bacterial* or anti-infective* or antibacterial* or antiinfective* or antiseptic*)

5. (MH "Infection+") OR TI ( infection* or SSI or SSIs ) OR AB ( infection* or SSI or SSIs )

The search was conducted up to 24-10-2023
