# Supplementary material for: Topical antimicrobial treatment of mesh for the reduction of surgical site infections after hernia repair: a systematic review and meta-analysis
Source: Hernia. 2024 May 9;28(3):691–700. doi: 10.1007/s10029-024-02987-0 (PMC11249405; doi:10.1007/s10029-024-02987-0)
Supplement: Supplementary file 2 — Supplementary file2 (DOCX 15 KB) [file 10029_2024_2987_MOESM2_ESM.docx]

**Topical Antimicrobial Treatment of Mesh for the Reduction of Surgical Site Infections after Hernia Repair**

A Systematic Review and Meta-Analysis

**Hernia**

# **Online Resource 2. Reasons for exclusion after full text review**

|  | **Study** | **Reason for exclusion** |
| --- | --- | --- |
| 1 | Roth 2022 [1] | Not assess PICO |
| 2 | Mirel 2022 [2] | Review article |
| 3 | Robinson 2021 [3] | Partial follow-up study from same population as Baker [4] |
| 4 | Warren 2019 [5] | Study protocol |
| 5 | Gogoladze 2016 [6] | Article not retrieveable |
| 6 | Cobb 2006 [7] | Not assess PICO |
| 7 | Tran 2006 [8] | Not assess PICO |
| 8 | Licheri 2004 [9] | Conference abstract |
| 1. Roth JS, Anthone GJ, Selzer DJ, Poulose BK, Pierce RA, Bittner JG, et al. (2022) Long-Term, Prospective, Multicenter Study of Poly-4-Hydroxybutyrate Mesh (Phasix Mesh) for Hernia Repair in Cohort at Risk for Complication: 60-Month Follow-Up. J Am Coll Surg 235(6):894-904.  2. Mirel S, Pusta A, Moldovan M, Moldovan S (2022) Antimicrobial Meshes for Hernia Repair: Current Progress and Perspectives. J Clin Med 11(3). https://doi.org/10.3390/jcm11030883  3. Robinson J, Sulzer JK, Motz B, Baker EH, Martinie JB, Vrochides D, Iannitti DA (2022) Long-Term Clinical Outcomes of an Antibiotic-Coated Non-Cross-linked Porcine Acellular Dermal Graft for Abdominal Wall Reconstruction for High-Risk and Contaminated Wounds. Am Surg 88(8):1988-95.  4. Baker EH, Lepere D, Lundgren MP, Greaney PJ, Ehrlich DA, Copit SE, et al. (2016) Early Clinical Outcomes of a Novel Antibiotic-Coated, Non-Crosslinked Porcine Acellular Dermal Graft after Complex Abdominal Wall Reconstruction. J Am Coll Surg 223(4):581-6. https://doi.org/10.1016/j.jamcollsurg.2016.05.022  5. Warren JA. (2019) Reducing INfection at the Surgical SitE With Antibiotic Irrigation During Ventral Hernia Repair (RINSE Trial) (RINSE). clinicaltrials.gov.  6. Gogoladze M, Kiladze M, Chkhikvadze T, Jiqia D (2016) [CLINICAL EVALUATION OF THE NEW ANTISEPTIC MESHES]. Georgian Med News (261):7-11.  7. Cobb WS, Paton BL, Novitsky YW, Rosen MJ, Kercher KW, Kuwada TS, Heniford BT (2006) Intra-abdominal placement of antimicrobial-impregnated mesh is associated with noninfectious fever. Am Surg 72(12):1205-8; discussion 8-9.  8. Tran DA, Truong QD, Nguyen MT (2006) Topical application of povidone-iodine solution (Betadine) in the management of giant omphaloceles. Dermatology 212 Suppl 1:88-90.  9. Licheri S, Erdas E, Pomata M, Pisano G, Daniele GM (2004) Femoral hernia repair with Bard Mesh Dart Plug. Chir Ital 56(5):705-10. | | |
