# Supplementary material for: Topical antimicrobial treatment of mesh for the reduction of surgical site infections after hernia repair: a systematic review and meta-analysis
Source: Hernia. 2024 May 9;28(3):691–700. doi: 10.1007/s10029-024-02987-0 (PMC11249405; doi:10.1007/s10029-024-02987-0)
Supplement: Supplementary file 3 — Supplementary file3 (DOCX 18 KB) [file 10029_2024_2987_MOESM3_ESM.docx]

**Topical Antimicrobial Treatment of Mesh for the Reduction of Surgical Site Infections after Hernia Repair**

A Systematic Review and Meta-Analysis

**Hernia**

**Online Resource 3. Secondary outcomes and SSI criteria**

| Study design | | Author, year | Type of mesh treatment | Hernia recurrence | Mesh infection | | | Pathogens | SSI criteria |
| --- | --- | --- | --- | --- | --- | --- | --- | --- | --- |
| RCT | | Musella (2001) | Gentamicin collagen tampon^1^ | NR | | NR | NR | | NR |
|  |  |  | No antimicrobial mesh treatment | NR | |  |  |  |  |
|  |  | Praveen (2009) | Soaked in Gentamicin | 1 (1%) | | NR | Staphylococcus aureus, Enterobacter, Staphyloccoccus coagulase negative | | CDC |
|  |  |  | No antimicrobial mesh treatment | 0 (0%) | |  |  |  |  |
|  |  | Şeker (2021) | Topical Gentamicin | NR | | NR | NR | | CDC |
|  |  |  | Topical Gentamicin | NR | |  |  |  |  |
|  |  |  | No antimicrobial mesh treatment | NR | |  |  |  |  |
|  |  | Yabanoğlu (2015) | Soaked in Vancomycin | NR | | NR | Pseudomonas aeruginosa, Staphylococcus, Staphylococcus aureus, enterococcus faecalis, Proteus mirabilis, E-coli | | CDC |
|  |  |  | No antimicrobial mesh treatment | NR | |  |  |  |  |
|  |  | Warren (2023) | Irrigated with Gentamicin + Clindamycin | NR | | NR | NR | | CDC |
|  |  |  | No antimicrobial mesh treatment | NR | |  |  |  |  |
| Observational | comparative | Fatula (2018) | Irrigated with Gentamicin | NR | | NR | NR | | NR |
|  |  |  | Irrigated with Gentamicin + Clindamycin | NR | |  |  |  |  |
|  |  |  | No antimicrobial mesh treatment | NR | |  |  |  |  |
|  |  | Kahramanca (2013) | Topical Rifampicin | 3 (2.2%) | | NR | Staphylococcus Aureus. Staphylococcus Epidermidis | | Clinical assessment |
|  |  |  | No antimicrobial mesh treatment | 1 (0.7%) | |  |  |  |  |
|  | Single arm | Baker (2016) | Coated with Rifampicin + Minocycline | 4 (5.4%) | | NR | NR | | NR |
|  |  | Drohan (2020) | CSAB with Vancomycin + Gentamicin^1^ | 1 (9%) | | NR | NR | | NR |
|  |  | IIahi (2023) | Coated with Rifampicin + Minocycline | 4 (6.8%) | | 1 (1.7%) | NR | | CDC |
|  |  | Schneeberger (2020) | Soaked in Povidone Iodine + Bacitracin + Gentamicin + Cefazolin | NR | | 2 (2.3%) | NR | | NR |
| Abbreviations: Centers for Disease Control and Prevention (CDC), Calcium Sulfate Antibiotic Beads (CSAB), Not Reported (NR), Randomized Controlled Trial (RCT), Surgical Site Infection (SSI). Note: ^1^ Placed in front of the mesh. | | | | | | | | | |
