# Supplementary material for: Topical antimicrobial treatment of mesh for the reduction of surgical site infections after hernia repair: a systematic review and meta-analysis
Source: Hernia. 2024 May 9;28(3):691–700. doi: 10.1007/s10029-024-02987-0 (PMC11249405; doi:10.1007/s10029-024-02987-0)
Supplement: Supplementary file 4 — Supplementary file4 (DOCX 19 KB) [file 10029_2024_2987_MOESM4_ESM.docx]

**Topical Antimicrobial Treatment of Mesh for the Reduction of Surgical Site Infections after Hernia Repair**

A Systematic Review and Meta-Analysis

**Hernia**

# **Online Resource 4. Statements on method of antimicrobial treatment of the mesh, mesh location and systemic antibiotic prophylaxis**

| **Study** | **Statements on method of antimicrobial treatment of the mesh** | **Statements on location of mesh placement n (%)** | **Statements on systemic antibiotic prophylaxis** |
| --- | --- | --- | --- |
| **Baker 2016** | Recently introduced to the market, XenMatrix AB Surgical Graft (XenAB; CR Bard, Inc. [Davol]) is a non-crosslinked porcine acellular dermal graft (NCPADG) with a tyrosine polymer coating containing the antibiotics rifampin and minocycline. Here we describe early clinical outcomes using XenAB  NCPADG for ventral hernia repair. | Extraperitoneal sublay 5 (6.8)  Intraperitoneal 26 (35.1)  Retrorectus sublay 9 (12.2)  Sandwich 2 (2.7)  Onlay 16 (21.6)  Other 14 (18.9) | NR |
| **Drohan 2020** | Patients undergoing single-stage hernia repair in contaminated fields with biologic porcine submucosa tissue matrix and the novel use of CSAB. We used Stimulan CSAB (Biocompsites Ltf.), which deliver high concentrations of vancomycin and gentamycin over a period of approximately 6 weeks, with minimal systemic absorption. CSAB were placed on top of the hernia graft with the fascia closed over top. They were also placed on top of the fascia in the subcutaneous space.  On postoperative day 1 the mean serum levels of the antibiotics were gentamycin 0.96 ± 0.8 mg/L and vancomycin 2.24 ± 0.8 mg/L. | …placed in the intraperitoneal abdominal position. | Ancef 2 g IV was administered preoperatively, and no systemic antibiotics were administered postoperatively. |
| **Fatula 2018** | Patients were divided into three groups. Patients in group 1 received no antibiotic irrigation. Group 2 patients received gentamicin 240 mg alone mixed in 500 mL of normal saline, with no standardized dwell time. Group 3 received a combination of gentamicin 240 mg and clindamycin 600 mg mixed in 500 mL normal saline. Antibiotic irrigation was used after mesh placement and fixation,  letting the irrigant dwell for three minutes before evacuating. | The majority of patients were repaired in a retromuscular  (RM) fashion with or without transversus  abdominis release (TAR) (n 4 624, 73.2%), whereas  the remainder were repaired as an onlay (n 4 43,  5.1%), intraperitoneal (n 4 4, 0.5%), or preperitoneal  (n4181, 21.2%). | Perioperative normothermia, appropriate hair removal, and antibiotic prophylaxis are standard. |
| **Ilahi 2023** | This study was a prospective, multicenter, single-arm clinical study designed to collect data on the performance of an antimicrobialcoated,noncrosslinked, AC-PDM (XenMatrix™AB Surgical Graft, C. R. Bard /Davol, Inc.) in a cohort of patients up to 24months postimplantation (ClinicalTrials.gov # NCT02691962 https://clinicaltrials.gov/ct2/show/NCT02691962). The PDM surface is coated with the antibacterial agents Rifampin and Minocycline in a bioresorbable L-Tyrosine succinate polymer carrier. | A total of n= 47 (62.7%) AC-PDM grafts were placed in  the retromuscular position (n= 28, 37.3% with component  separation technique (CST) and n=19, 25.3% without CST).  A total of n=28 (37.3%) AC-PDM grafts were placed in the  intraperitoneal position (n=12, 16.0% with CST and n= 16,  21.3% without CST). | Prophylactic antibiotics were administered according to participating hospital protocol, and patients underwent open ventral hernia repair. |
| **Kahramanca 2013** | The 278 study patients were divide into two groups according to their antibiotic use; 144 patients who did not receive any prophylactic systemic antibiotics constituted Group 1, 134 patients who received 250 mg topical Rifampicin over the mesh based on surgeon’s preference constituted Group 2. | NR | See Statements on method of antimicrobial treatment of the mesh (left column). |
| **Musella 2001** | Patients in the control group n = 294 had a standard surgical treatment and they were compared with another of 301 patients in whom an absorbable collagen tampon (Collatamp G1Innocol, Saal/Donau, Germany) treated with gentamicin (Fig. 1a), was placed in front of the prosthetic mesh (Fig. 1b), tailored to the patient and covered by the sutured aponeurosis of the external oblique muscle. | the prosthetic mesh, tailored  to the patient and covered by the sutured aponeurosis of  the external oblique muscle. | Patients in both groups were given long-acting cephalosporins, ceftriaxone 2 g systemically, 1 hour before and 12 hours after the intervention, at home if discharged from hospital. |
| **Praveen 2009** | Patients were randomised to receive either IV 240 mg gentamicin diluted into 10 mL saline, given during induction, or 160 mg gentamicin used as local antibiotic. The polypropylene mesh was soaked in 160 mg of gentamicin diluted in 250 mL of normal saline and the inguinal wound was irrigated with the gentamicin solution. | Lichtenstein (preperitoneal) | See Statements on method of antimicrobial treatment of the mesh (left column). |
| **Schneeberger 2020** | The mesh was removed from its packaging only immediately before use (ensuring that the mesh had minimal exposure to the surrounding environment) and dipped in the antibiotic solution and povidone-iodine. The mesh never touched the Mayo stand, drapes, sponges, or other instruments at any time. 6. The surgeon placed the mesh in the appropriate abdominal wall position while avoiding skin contact. 7. The incision was again thoroughly irrigated with the antibiotic solution. | Onlay 1 (1.1)  Open preperitoneal sublay 1 (1.1)  Open intraperitoneal sublay 20 (22.8)  Open retromuscular sublay (no TAR) 30 (34.1)  Open retromuscular sublay (with TAR) 36 (40.9) | NR |
| **Şeker 2021** | Patients in G1 group received 1 g cefazolin (cefazolin sodium; Eczacibasi, Istanbul, Turkey) by IV bolus injection. In G2, gentamicin (gentamicin sulfate 80 mg 2%; I, E Ulugay, Istanbul, Turkey) was applied topically to surgical field just after the mesh implantation. In G3, patients received 1 g cefazolin and topical gentamicin. The anesthesiologist administered IV medication when the patient entered the operating room or at least, before the induction of anesthesia. None of the patients in either group was prescribed for any additional antibiotics. | Lichtenstein (preperitoneal) | See Statements on method of antimicrobial treatment of the mesh (left column). |
| **Warren 2024** | After completion of the retromuscular dissection and TAR (if required), closure of the posterior sheath, and mesh placement in the RM space, final determination for inclusion/exclusion was made by the operating surgeon. Included patients were randomized to dual-antibiotic irrigation consisting of 240 mg gentamicin þ600 mg clindamycin in 500 ml saline (G þ C) or saline irrigation alone. The irrigant was instilled in the RM space, filling the compartment up to the level of the skin and completely covering the implanted mesh. This was allowed to dwell for 3 min and then evacuated. | Retromuscular | Preoperative intravenous antibiotics were used in all patients with antibiotic selection  based on standard practice and antibiogram of each site. |
| **Yabanoğlu 2015** | In group 1 (n = 26), the synthetic mesh was soaked in 0.9% saline solution  15 minutes before implantation. In group 2 (n = 26), the synthetic mesh was soaked in Vancomycin solution (10 mg/mL) 15 minutes before implantation. | Onlay 5 (9.6)  Sublay 47 (90) | All patients received antibiotic prophylaxis prior to the operation, before entering the operating room (2 g first-generation cephalosporin (Sefazol, Mustafa Nevzat, Istanbul, Turkey). |
| Acellular porcine dermal matrix. (AC-PDM), Calcium Sulfate Antibiotic Beads (CSAB), Not Reported (NR), porcine dermal matrix (PDM) | | | |
