# Supplementary material for: Topical antimicrobial treatment of mesh for the reduction of surgical site infections after hernia repair: a systematic review and meta-analysis
Source: Hernia. 2024 May 9;28(3):691–700. doi: 10.1007/s10029-024-02987-0 (PMC11249405; doi:10.1007/s10029-024-02987-0)
Supplement: Supplementary file 5 — Supplementary file5 (DOCX 87 KB) [file 10029_2024_2987_MOESM5_ESM.docx]

**Topical Antimicrobial Treatment of Mesh for the Reduction of Surgical Site Infections after Hernia Repair**

A Systematic Review and Meta-Analysis

**Hernia**

# **Online Resource 5. Elaborate Newcastle-Ottawa Scale and risk of bias and assessment**

| **Quality assessment, according to the Newcastle-Ottawa Scale** | | | | | | | | | | |
| --- | --- | --- | --- | --- | --- | --- | --- | --- | --- | --- |
| The Newcastle-Ottawa Scale[1] contains 8 items within 3 domain with a maximum score of nine stars. A study can be awarded a maximum of one star for each numbered item within the selection and outcome domains. A maximum of two stars can be given for comparability.  Good quality: study with 3 or 4 stars in selection domain AND 1 or 2 stars in comparability domain AND 2 or 3 stars in outcome domain.  Fair quality: 2 stars in selection domain AND 1 or 2 stars in comparability domain AND 2 or 3 stars in outcome domain.  Poor quality: 0 or 1 star in selection domain OR 0 stars in comparability domain OR 0 or 1 stars in outcome domain. | | | | | | | | | | |
|  | Selection | | | | Comparability | Outcome | | | | |
|  | Representativeness of the exposed cohort | Selection of the non-exposed cohort | Ascertainment of exposure | Demonstration that outcome of interest was not present at start of study | Comparability of cohorts on the basis of the design or analysis controlled for confounders | Assessment of outcome | Follow-up long enough for outcomes to occur | Adequacy of follow-up of cohorts | Stars | Quality assess-ment |
| Baker 2016 | **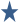** | - | **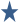** | **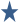** | - | **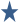** | **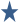** | **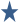** | 6 | Poor |
| Drohan 2020 | **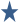** | **-** | **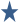** | **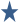** | **-** | **-** | **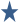** | **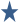** | 5 | Poor |
| Fatula 2018 | **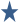** | **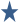** | **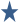** | **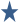** | **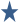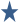** | **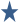** | **-** | **-** | 7 | Poor |
| IIahi 2023 | **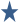** |  | **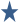** | **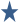** | **-** | **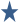** | **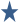** | **-** | 5 | Poor |
| Kahramanca 2013 | **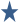** | **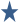** | **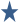** | **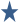** | **-** | **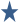** | **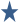** | **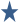** | 7 | Poor |
| Schneeberger 2020 | **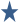** | **-** | **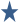** | **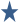** | **-** | **-** | **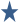** | **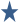** | 5 | Poor |
| [1] S. B. Wells G, O'Connell D, Peterson J, Welch V, Losos M, Tugwell P. "The Newcastle-Ottawa Scale (NOS) for assessing the quality of nonrandomised studies in meta-analyses. 2013." <http://www.ohri.ca/programs/clinical_epidemiology/oxford.asp> (accessed November 10, 2023). | | | | | | | | | | |


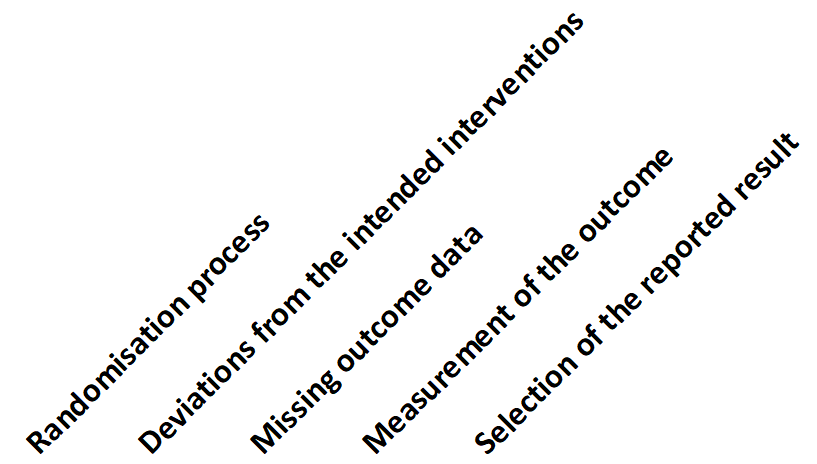


| **Study**  Musella 2001 |  |  |  |  |  | **Overall** |  |  |  |
| --- | --- | --- | --- | --- | --- | --- | --- | --- | --- |
| Praveen 2009 |  |  |  |  |  |  |  |  | Low risk |
| Şeker 2021 |  |  |  |  |  |  |  |  | Some concerns |
| Waren 2023 |  |  |  |  |  |  |  |  | High risk |
| Yabanoğlu 2015 |  |  |  |  |  |  |  |  |  |
