# Supplementary material for: Topical antimicrobial treatment of mesh for the reduction of surgical site infections after hernia repair: a systematic review and meta-analysis
Source: Hernia. 2024 May 9;28(3):691–700. doi: 10.1007/s10029-024-02987-0 (PMC11249405; doi:10.1007/s10029-024-02987-0)
Supplement: Supplementary file 6 — Supplementary file6 (DOCX 25 KB) [file 10029_2024_2987_MOESM6_ESM.docx]

**Topical Antimicrobial Treatment of Mesh for the Reduction of Surgical Site Infections after Hernia Repair**

A Systematic Review and Meta-Analysis

**Hernia**

**Online Resource 6. GRADE Assessment**

The Grading of Recommendation, Assessment, Development and Evaluation (GRADE) approach was used to grade certainty of evidence using a minimally contextualized approach on the following five domains: risk of bias, inconsistency, indirectness, imprecision and publication bias [1]. The minimally important difference was defined as 1.15% based on the default for appreciable benefit and harm of 25% and the SSI incidence of 4.6% in data of present meta-analysis of included RCTs for patients without antibiotic mesh treatment [2]. We evaluated imprecision taking the minimally important differences into account. In case of large effects, the optimal information size approach was used by calculating the ratio of the upper to the lower boundary of the confidence interval with a threshold for downgrading of 2.5 [3].

Since all included studies are randomized controlled trials, the starting certainty of evidence was high. Downgrading can be necessary due to the following reasons:

- Risk of bias

Of the 4 included studies in the meta-analysis, there was one study with high risk of bias. We performed a sensitivity analysis excluding the study with high risk of bias. The results were comparable to the overall analysis (RR 0.91; 95% CI 0.32-2.58), and downgrading for risk of bias was not necessary.

- Inconsistency
  For inconsistency -1 downgrade was necessary (*I*^2^ = 50%, τ^2^ = 0.5001).
- Indirectness
  No downgrade was needed, since the body of evidence of the studies does represent the PICO elements of interest [4].
- Imprecision
  The CI overlapped the thresholds of interest. Therefore, we downgraded two levels for imprecision.
- Publication bias
  Rating down one level for publication bias was necessary because the evidence consists of a number of small studies.

|  | **Certainty assessment** | | | | | | | **No of patients** | | **Effect** | | **Certainty** |
| --- | --- | --- | --- | --- | --- | --- | --- | --- | --- | --- | --- | --- |
|  | No of studies | Study design | Risk of bias | Inconsistency | Indirectness | Imprecision | Publication bias | Topical AB | Control | Relative (95% CI) | Absolute (95% CI) |  |
| SSI | 4 | RCT | Not serious | Serious  (-1 downgrade) | Not serious | Serious  (-2 downgrade) | Serious  (-1 downgrade) | 19/520 (3.7%) | 24/519 (4.6%) | 0.76 (0.27-2.09) | 10 fewer per 1000  (from 15 fewer to 34 more) | ⨁◯◯◯ very low |
| AB = antibiotics; CI = confidence interval; GRADE = Grading of Recommendations, Assessment, Development and Evaluation; PICO = population, intervention, comparison and outcomes; RCT = randomized controlled trial; RR = relative risk; SSI = surgical site infection | | | | | | | | | | | | |

**References**

1. Schünemann HB JG, G.; Oxman, A. (2013) GRADE Handbook for grading quality of evidence and strength of recommendations. Updated October 2013. <https://gdt.gradepro.org/app/handbook/handbook.html>. Accessed 29 November 2023.

2. Zeng L, Brignardello-Petersen R, Hultcrantz M, Siemieniuk RAC, Santesso N, Traversy G, et al. (2021) GRADE guidelines 32: GRADE offers guidance on choosing targets of GRADE certainty of evidence ratings. J Clin Epidemiol 137:163-75. <https://doi.org/10.1016/j.jclinepi.2021.03.026>

3. Zeng L, Brignardello-Petersen R, Hultcrantz M, Mustafa RA, Murad MH, Iorio A, et al. (2022) GRADE Guidance 34: update on rating imprecision using a minimally contextualized approach. J Clin Epidemiol 150:216-24. <https://doi.org/10.1016/j.jclinepi.2022.07.014>

4. Zhang Y, Alonso-Coello P, Guyatt GH, Yepes-Nuñez JJ, Akl EA, Hazlewood G, et al. (2019) GRADE Guidelines: 19. Assessing the certainty of evidence in the importance of outcomes or values and preferences-Risk of bias and indirectness. J Clin Epidemiol 111:94-104. <https://doi.org/10.1016/j.jclinepi.2018.01.013>
